# Diabetes as a Risk Factor for Sarcopenia in Patients with MASH-Related Cirrhosis

**DOI:** 10.3390/jcm14248691

**Published:** 2025-12-08

**Authors:** Shinya Sato, Hiroaki Takaya, Tadashi Namisaki, Tatsuya Nakatani, Jun-ichi Hanatani, Yuki Tsuji, Koh Kitagawa, Norihisa Nishimura, Kosuke Kaji, Hitoshi Yoshiji

**Affiliations:** Department of Gastroenterology, Nara Medical University, Kashihara 634-8521, Japan; htky@naramed-u.ac.jp (H.T.); tadashin@naramed-u.ac.jp (T.N.); k182324@naramed-u.ac.jp (T.N.); k136081@naramed-u.ac.jp (J.-i.H.); tsujih@naramed-u.ac.jp (Y.T.); kitagawa@naramed-u.ac.jp (K.K.); nishimuran@naramed-u.ac.jp (N.N.); kajik@naramed-u.ac.jp (K.K.); yoshijih@naramed-u.ac.jp (H.Y.)

**Keywords:** metabolic dysfunction-associated steatotic liver disease, liver cirrhosis, sarcopenia, diabetes mellitus

## Abstract

**Objectives:** Metabolic dysfunction-associated steatohepatitis (MASH) is a leading cause of cirrhosis within the spectrum of metabolic dysfunction-associated steatotic liver disease (MASLD). However, the prognostic impact of diabetes mellitus (DM) in MASH-associated cirrhosis remains unclear. This study aimed to compare clinical outcomes between cirrhotic patients with and without DM. **Methods:** Patients with MASH-related cirrhosis were stratified into DM (DM-MASH) and non-DM (non-DM MASH) groups. The diagnosis of MASH was based on histological evidence of steatohepatitis with underlying metabolic dysfunction. The non-DM group included both obese individuals and lean/normal-weight individuals with ≥1 metabolic risk factors. Mortality, liver-related events (LREs; ascites, variceal bleeding, encephalopathy, and hepatocellular carcinoma), and sarcopenia were compared using Kaplan–Meier analysis, log-rank tests, and Fisher’s exact test. Risk factors for sarcopenia were assessed using logistic regression. **Results:** Median survival was significantly shorter in DM-MASH patients compared to non-DM MASH patients (1523 vs. 2618 days; *p* < 0.05). The incidence of LREs during follow-up was also higher in the DM-MASH group. The prevalence of sarcopenia was significantly greater among DM-MASH patients (36.1% vs. 19.7%; *p* < 0.05). In multivariate analysis, DM emerged as an independent predictor of sarcopenia in patients with MASH-related cirrhosis. **Conclusions:** DM is associated with worse outcomes in MASH-driven cirrhosis, including increased sarcopenia and reduced survival. DM may serve as a prognostic marker for identifying high-risk patients with MASH-associated cirrhosis.

## 1. Introduction

Liver cirrhosis contributes significantly to the morbidity and mortality of patients with chronic liver diseases [1]. Cirrhosis is a diffuse process characterized by tissue fibrosis and the conversion of the normal liver architecture into structurally abnormal nodules [2]. This process significantly impairs liver function. Cirrhosis can lead to hepatocellular carcinoma (HCC) and hepatic decompensation, which includes conditions like ascites, hepatic encephalopathy, variceal bleeding, and sarcopenia [1,3,4,5]. Cirrhosis is a major cause of global mortality, accounting for approximately 2.4% of global deaths in 2019 [6].

Owing to the rising prevalence of obesity and increased alcohol consumption on the one hand, and improvements in the management of hepatitis B virus and C virus infections on the other, the epidemiology of cirrhosis is changing. The major etiologies of liver cirrhosis in Japan are reported to have been shifting from viral hepatitis to nonviral chronic liver diseases. The percentage of metabolic dysfunction-associated steatohepatitis (MASH) associated cirrhosis has been increasing from 4.7% in 2008–2010 to 14.8% in 2018–2021 [7].

In June 2023, a multi-society Delphi consensus statement on a new fatty liver disease nomenclature was published, introducing the term metabolic dysfunction-associated steatotic liver disease (MASLD) and effectively retiring the term nonalcoholic fatty liver disease (NAFLD) [8] because the latter did not adequately reflect the involvement of metabolic dysfunction. However, MASLD/MAFLD emphasizes the definition of “metabolic dysfunction-associated steatotic liver disease,” providing a more accurate representation of the disease’s underlying pathophysiology.

MASH progressing to cirrhosis often coexists with type 2 diabetes mellitus (T2DM), a condition that exacerbates systemic inflammation, insulin resistance, and fibrogenesis—factors implicated in both hepatic and muscular deterioration. Sarcopenia is notably prevalent in MASLD/MASH and cirrhosis, with estimates ranging from about 20% to 40%, and has been established as an independent predictor of disease progression and mortality [9]. Furthermore, T2DM not only increases the prevalence of MASLD but also contributes to sarcopenia through impaired insulin signaling, reduced anabolic hormone activity, and chronic inflammation that accelerate muscle loss [10]. Recent evidence further suggests that sarcopenia is particularly common in MASH and closely associated with fibrosis severity and adverse outcomes [11]. Taken together, these interactions indicate that diabetes may potentiate sarcopenic burden in MASH cirrhosis, underscoring a critical gap in understanding its mechanistic role in worsening prognosis.

A challenge in treating patients with MASLD is their highly diverse population as the previous criteria (NAFLD/NASH) were exclusionary. The new criteria for MASLD/MASH emphasize DM, obesity, and metabolic abnormalities, allowing patients to be divided into subgroups for treatment rather than being treated as a single homogeneous group. DM is associated with an increased risk of advanced fibrosis, cirrhosis-related complications, and liver disease mortality. In this study, our primary objective was to clarify the prognostic impact of diabetes mellitus on MASH-related cirrhosis by comparing mortality, liver-related events, and sarcopenia between patients with and without DM.

## 2. Materials and Methods

### 2.1. Study Design

In this single-center retrospective study conducted at Nara Medical University (Kashihara, Nara, Japan), we enrolled 169 Japanese patients with MASH-driven cirrhosis who received treatment at the Department of Gastroenterology in Nara Medical University Hospital between March 2013 and December 2023. The protocol was reviewed and approved by the Institutional Review Board of Nara Medical University. In accordance with institutional policy for retrospective studies, the requirement for written informed consent was waived. Instead, an opt-out procedure was implemented, whereby patients were informed of the study, and those who declined participation were excluded. The patients’ laboratory data were obtained at the time of diagnosis of MASH-related cirrhosis.

### 2.2. Eligibility Criteria

We enrolled patients with cirrhosis aged >20 years who were diagnosed on the basis of physical examinations, laboratory data, imaging data, and sometimes biopsy. Our exclusion criteria were as follows: (1) the presence of other causes of cirrhosis except for MASH; (2) hepatocellular carcinoma; (3) malignancy, other than hepatocellular carcinoma; (4) history of gastrectomy; (5) severe infection, respiratory, renal, and/or cardiac diseases; (6) not undergoing computed tomography. In addition, patients presenting with moderate-to-severe ascites or overt hepatic encephalopathy were excluded from the study. Only patients with minimal ascites that was well controlled were eligible for inclusion.

### 2.3. Patients and Assessment of MASH-Driven Cirrhosis

MASH-driven cirrhosis is defined as the presence of cirrhosis in a patient with a history of MASLD. A new definition of steatosis, MASLD, has been proposed by international expert panels [12]. The diagnosis of MASLD requires the presence of steatosis (by histology or imaging) in combination with at least one cardiometabolic risk factor (CMRF). Fatty liver can be evaluated either by imaging modalities, blood biomarkers/scores, or liver histology. Overweight was defined as body mass index (BMI) ≥ 23 kg/m^2^ for Asians [13], and type 2 DM was defined as HbA1c ≥ 6.5% or specific drug treatment for the condition. CMRF was defined as the presence of at least one of the following metabolic risk abnormalities: (1) BMI ≥ 23 kg/m^2^ or waist circumference ≥ 90 cm and 80 cm in males and females, respectively; (2) HbA1c ≥ 5.7% or T2DM or treatment for T2DM (3) blood pressure ≥ 130/85 mmHg or specific drug treatment for the condition; (4) plasma triglyceride level ≥ 150 mg/dl or specific drug treatment for the condition; (5) plasma high-density lipoprotein cholesterol level ≤ 40 mg/dL and 50 mg/dL in males and females, respectively [8].

To assess the prognostic importance of DM, MASH-driven cirrhotic patients were regrouped into MASH with DM (DM-MASH) and without DM (non-DM MASH; obese-MASH and metabolic dysfunction-MASH). We compared these two groups based on mortality, LREs, and other factors such as sarcopenia. LREs include hepatic decompensation (ascites, hemorrhagic varices, and encephalopathy) and hepatocellular carcinoma. Kaplan–Meier survival analyses and log-rank tests were used to compare the mortality and incidence rates of LREs between the two groups.

### 2.4. Biochemical Analyses

A blood sample was obtained after an overnight fast, and the following biochemical parameters were measured: hemoglobin, platelet count, aspartate aminotransferase (AST), alanine aminotransferase (ALT), total protein, albumin, triglycerides, total cholesterol, cholinesterase, total bilirubin, ammonia, prothrombin time, and hemoglobin A1c (HbA1c). Liver functional reserve was evaluated using the Child–Pugh score. This scoring system is based on laboratory parameters, including serum albumin, prothrombin time, and total bilirubin, as well as clinical findings such as ascites and hepatic encephalopathy. The fibrosis-4 (FIB-4) index is a noninvasive scoring system used to assess liver fibrosis. The FIB-4 index is calculated as follows: Fibrosis-4 (FIB-4) index: FIB-4 = [AST (IU/L) × age (years)]/[ALT(IU/L)^1/2^ × platelets (10^3^/μL)] [14].

### 2.5. Assessment of Sarcopenia

Sarcopenia was diagnosed using only the skeletal muscle mass index (SMI) as this study was retrospective, meaning we could not obtain data about grip strength. SMI was measured as the muscle mass of the cross-sectional area of the skeletal muscles (cm^2^) at the lumbar vertebra (L3) assessed using computed tomography (CT), divided by the square of the height [L3 SMI = cm^2^/m^2^ [15]. The skeletal muscle area, composed of the transversus abdominis, the quadratus lumborum, the psoas major, the erector spinae, the rectus abdominis, the internal oblique, and the external oblique, was measured by the cross-sectional CT images at L3 using Slice-O-Matic software (version 5.0; Tomovision). SMI ≤ 42.0 cm^2^/m^2^ for men and ≤38.0 cm^2^/m^2^ for women was defined as a low SMI [16].

### 2.6. Statistical Analyses

Statistical analyses were performed using EZR (Saitama Medical Center, Jichi Medical University, Shimotsuke, Tochigi, Japan), a graphical user interface for R (version 4.1.2; The R Foundation for Statistical Computing, https://www.r-project.org). EZR is a modified version of the R commander version 2.7-1 that includes statistical functions frequently used in biostatistics [17]. Quantitative data are presented as medians and interquartile ranges while categorical data are presented as frequencies and percentages. Between-group differences were analyzed using the chi-square test or Mann–Whitney U test. The survival rate was estimated with the Kaplan–Meier method and between-group comparisons were performed using the log-rank test. Hazard ratios (HRs) and 95% confidence intervals (CIs) were estimated with a Cox proportional-hazards model. Correlations were evaluated using scatter plots. Univariate and multivariate analyses were performed using logistic regression to identify factors associated with sarcopenia. Furthermore, we performed a multivariate analysis adjusted using model fit estimation based on the Akaike Information Criterion (AIC) selection. Two-tailed *p*-values < 0.05 were considered statistically significant.

## 3. Results

### 3.1. Baseline Characteristics

We enrolled 169 patients with MASH-driven cirrhosis in this retrospective study. The baseline characteristics of the study population are shown in Table 1. The mean age was 70 years. The patients consisted of 77 males (45.5%) and 92 females (54.5%). In addition, there were 58 patients with Child–Pugh class B or C among them. Of these, 108 (63.9%) patients were diagnosed with diabetes (DM-MASH). Total protein and triglyceride levels were higher in subjects with diabetes than in those without diabetes. (*p* = 0.04, 0.02, respectively). The clinical characteristics and blood biochemistry data did not differ significantly between the non-DM and DM groups, except for HbA1c (Table 1).

### 3.2. The Impact of Diabetes on the Prognosis of Patients with MASH-Driven Cirrhosis

**Figure 1:**
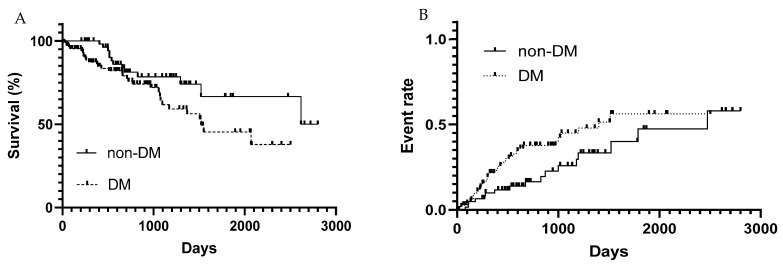
(**A**) Survival rates of MASH patients in the non-DM and DM groups. The survival rate of MASH patients was significantly lower in the DM group than in the non-DM group (*p* = 0.04). (**B**) The incidence rate of LREs in MASH patients in the non-DM and in DM groups. The survival rate was estimated using the Kaplan–Meier method and between-group comparisons were performed using the log-rank test. The incidence rate of LREs in MASH patients with DM was higher than that in MASH patients without DM (*p* = 0.04). MASH, Metabolic Dysfunction-Associated Steatohepatitis; DM, diabetes mellitus; LRE, liver-related events.

**Figure 2:**
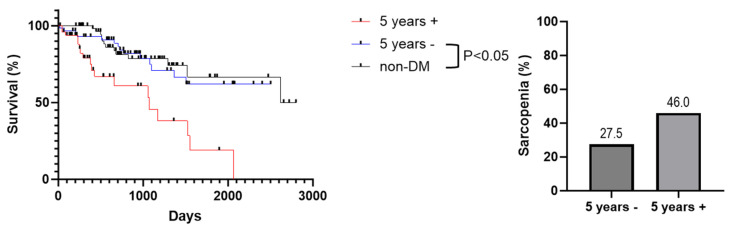
**Survival rates and prevalence of sarcopenia in MASH patients stratified by diabetes status and disease duration.** Survival rates of MASH patients were compared among the non-DM group, the ≥5-year DM duration group (5 years +), and the <5-year DM duration group (5 years −). Although no significant difference was observed between the non-DM and 5 years − groups, both demonstrated significantly longer survival compared with the 5 years + group. In addition, the prevalence of sarcopenia was higher in the 5 years + group than in the 5 years − group.

**Figure 3:**
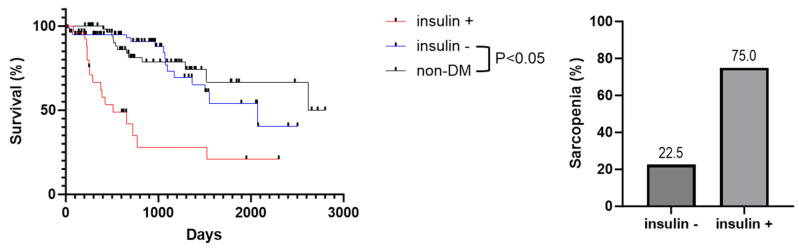
**Survival rates and prevalence of sarcopenia according to diabetes status and insulin use in patients with MASH.** Survival rates were compared among the non-DM, insulin-positive, and insulin-negative groups. Although no significant difference was observed between the non-DM and insulin-negative groups, both demonstrated significantly longer survival compared with the insulin-positive group. Furthermore, the prevalence of sarcopenia was higher in the insulin-positive group than in the insulin-negative group.

To compare survival rates between MASH patients with or without DM, we utilized the Kaplan–Meier method and the log-rank test. The survival rate of MASH patients was significantly lower in the DM group than in the non-DM group (Figure 1A). The one-year, three-year, and five-year survival rates in the DM group were 87.3%, 64.3%, and 45.3%, respectively, whereas those of the patients in the non-DM group were 100%, 78.3%, and 66.6%, respectively. The median survival time of DM-MASH patients was shorter than that of MASH without DM patients (1523 days [19–2402] vs. 2618 days [207–2801], *p* < 0.05, HR, 1.873; 95% Cl, 1.038–3.377). Next, we assessed the incidence of LREs as it directly leads to a poor prognosis. We compared the LRE incidence rates between groups with the Kaplan–Meier method and the log-rank test (Figure 1B). The LRE incidence rate in MASH with DM cases was higher than that in MASH without DM cases during the follow-up period (*p* = 0.04). MASH patients with DM were thus shown to have a worse prognosis than those without DM. Furthermore, among patients with diabetes, we examined disease duration (≥5 years vs. <5 years) and insulin use. Kaplan–Meier survival analysis showed that the non-DM group did not differ significantly from the <5 years group, but a significant difference was observed compared with the ≥5 years group. (Figure 2) Similarly, when comparing non-DM, insulin-treated, and non–insulin-treated groups, the non-DM group did not differ significantly from the non–insulin-treated group, whereas a significant difference was observed compared with the insulin-treated group (Figure 3).

### 3.3. Prevalence of Sarcopenia in MASH Patients With and Without DM

Sarcopenia is one of the most common complications related to the survival of patients with cirrhosis [18]. Among 169 patients with MASH-driven cirrhosis, 51 (30.1%) were diagnosed with sarcopenia, as diagnosed on abdominal CT at the level of T3 by the skeletal muscle area. Grip strength data from patients are not available as this study is retrospective. Comparing the clinical characteristics between patients with and without sarcopenia, we found that age, Child–Pugh class, hemoglobin, albumin, total cholesterol, cholinesterase, FIB-4 index, and BMI differed significantly between the two groups, as detailed in Table 2. The FIB-4 index can be measured by a blood-based diagnostic test for underlying fibrosis, which helps in determining the MASH status [19].

**Figure 4:**
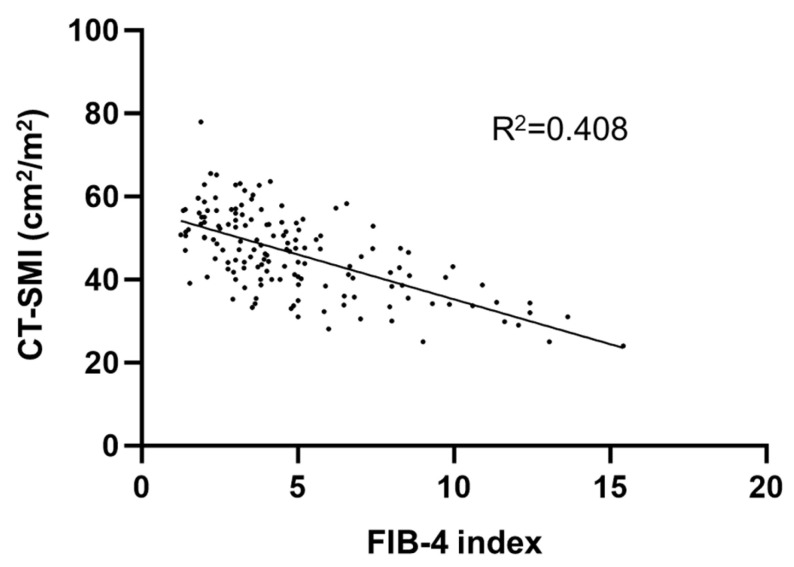
Correlation between CT-SMI (cm^2^/m^2^) and the FIB-4 index. This correlation was evaluated using a scatterplot. CT-SMI was negatively correlated with the FIB-4 index (R^2^ = −0.408, *p* < 0.001).

As there was a significant prognostic difference between MASH patients by DM status, we compared the prevalence of sarcopenia between the two groups. The prevalence of sarcopenia in MASH patients was higher in the DM group (36.1%,) than in the non-DM group (19.7%). In addition, we examined the prevalence of sarcopenia in patients with diabetes according to disease duration and insulin use. Among patients with a disease duration of ≤5 years, the prevalence of sarcopenia was 27.5%, whereas it was 46.0% in those with a duration of >5 years. (Figure 2) Furthermore, among patients with diabetes, the prevalence of sarcopenia was 22.5% in those not receiving insulin, compared with 75.0% in those treated with insulin (Figure 3). Although there was no difference in BMI and Child–Pugh class, which is associated with sarcopenia in cirrhotic patients, a higher incidence of sarcopenia was observed in the DM group. Further, we assessed CT-SMI and FIB-4 index in all patients. Pearson’s correlation analysis found that CT-SMI was negatively correlated with the FIB-4 index (R^2^ = −0.408, *p* < 0.001) (Figure 4). The worse prognosis of DM MASH patients compared to non-DM MASH patients is thus associated with sarcopenia occurrence.

### 3.4. Predictors of Sarcopenia in Patients with Cirrhosis Due to MASH

To determine the predictors of sarcopenia among patients with MASH, we initially selected nine variables based on the results presented in Table 2 for the logistic regression analysis. The optimal cut-off value of age for predicting sarcopenia was 75 years, and its area under the receiver operating characteristic curve, sensitivity, and specificity were 65.3%, and 58.8%, respectively. In the same manner, the optimal cut-off values were B or C for the Child–Pugh class,11.8 g/dL in hemoglobin, 3.5 g/dL for serum albumin levels, 144 mg/dL for the total cholesterol level, 105 IU/L for the cholinesterase level, 5 for the FIB-4 index, and 25 kg/m^2^ for BMI. However, the reference range for cholinesterase is 234–493 U/L for men and 200–452 U/L for women in Japan, and 105 IU/L is an abnormally low value. Therefore, the cut-off value was set at 200 IU/L for the cholinesterase level.

The univariate analysis confirmed that age ≥ 75, hemoglobin ≥ 11.8 g/dL, albumin ≥ 3.5 g/dL, total cholesterol ≥ 144 mg/dL, cholinesterase ≥ 200 IU/L, FIB-4 index ≥ 5, BMI ≥ 25 kg/m^2^ and DM were significantly associated with sarcopenia in MASH patients with liver cirrhosis, except for Child–Pugh class ≥ B, which was marginally associated in our statistical analysis (Table 3). Cholinesterase was initially analyzed using a cut-off of 105 IU/L based on our institutional reference value. However, as this threshold was considered relatively low, we re-analyzed the data using 200 IU/L as a clinically more meaningful cut-off.

Our multivariate logistic regression analysis incorporating these nine factors found FIB-4 index (cOR = 8.6, 95% CI = 3.24–22.8; *p* < 0.0001), BMI (cOR = 0.13, 95% CI = 0.05–0.32; *p* < 0.0001), and diabetes (cOR = 4.38, 95% CI = 1.55–12.3; *p* = 0.005) to be significantly associated with sarcopenia in these patients (Table 3). Furthermore, when we performed a multivariate analysis adjusted using model fit estimation based on the Akaike Information Criterion (AIC) selection, four factors were extracted. Among these, the FIB-4 index (aOR = 8.1, 95% CI = 3.35–19.6; *p* < 0.0001), diabetes (aOR = 3.67, 95% CI = 1.41–9.56; *p* = 0.007), and BMI (aOR = 0.114, 95% CI = 0.04–0.24; *p* < 0.0001) were significantly associated with sarcopenia. Liver fibrosis and BMI are reported to be associated with sarcopenia in liver diseases. In addition, diabetes was identified as an important determinant of sarcopenia in patients with MASH-related cirrhosis through the multiple logistic regression analysis. In this study, we identified a list of predictors of sarcopenia in patients with MASH-related cirrhosis.

## 4. Discussion

The population of patients with MASLD is diverse. The new criteria for MASLD/MASH are not exclusionary, emphasizing DM, obesity, and metabolic abnormalities as key traits. By categorizing MASLD/MASH cirrhosis patients by these traits, we can identify high-risk groups with poor prognoses. In this study, the analysis was conducted by comparing DM vs. non-DM groups. This approach was chosen because the primary aim of the study was to evaluate the impact of diabetes on sarcopenia and prognosis in MASH cirrhosis. Moreover, we compared the effects of MASH-driven cirrhosis with and without DM on mortality, liver-related events (LREs), and other factors such as sarcopenia. The median survival time of patients with DM-MASH was shorter than that of patients with non-DM MASH. Moreover, the incidence rate of LREs in MASH with DM cases was higher than that in MASH without DM cases during the follow-up period. Interestingly, the prevalence of sarcopenia was higher in MASH with DM than in MASH without DM.

The FIB-4 index and sarcopenia have attracted attention in chronic liver disease. A high FIB-4 index, indicating advanced liver fibrosis, is associated with an increased risk of sarcopenia [20]. This is thought to be due to impaired liver function affecting the nutritional status and metabolic processes, leading to a deficiency of essential factors required for muscle synthesis and maintenance. BMI is used as an indicator of nutritional status. A meta-analysis has shown that the sarcopenia group has a significantly lower BMI compared to the non-sarcopenia group [21]. Naturally, in patients with liver cirrhosis, a low BMI is associated with a higher risk of sarcopenia. In patients with MASH, sarcopenic obesity is also a concern, and even those with a high BMI are at risk of developing sarcopenia [22]. However, since the present study focused on patients with liver cirrhosis, it is reasonable that the results indicate that patients with a low BMI and poor nutritional status were more predisposed to sarcopenia. Furthermore, cholinesterase levels differed significantly according to the presence or absence of sarcopenia. As cholinesterase reflects both nutritional status and hepatic functional reserve in patients with cirrhosis, it represents an important clinical biomarker.

It has been reported that diabetes was a significant factor in the progression of MASH to liver cirrhosis [23]. However, there are not many studies discussing the significance of diabetes in liver cirrhosis derived from MASH. This study suggests that even in the advanced stage of liver cirrhosis, diabetes remains an important factor leading to sarcopenia. Therefore, patients with both MASH-driven liver cirrhosis and diabetes require particularly strict follow-up not only for glycemic control and muscle mass but also for appropriate dietary management.

In patients with cirrhosis, type 2 diabetes mellitus (T2DM) is commonly observed, with the highest prevalence seen in association with MASLD, the primary cause of chronic liver disease [24]. Globally, T2DM and cirrhosis contribute to approximately 5 million and 1.2 million deaths annually, respectively [25]. Diabetes, an independent poor prognostic factor in patients with cirrhosis, is associated with the occurrence of major complications such as ascites, encephalopathy, and infections [26]. However, the occurrence of cirrhosis can impact the identification and treatment of T2DM. A forward-looking community-based study in Singapore, involving 63,275 patients, revealed that diabetes was associated with a higher risk of cirrhosis-related mortality, especially in cases of cirrhosis not related to viral hepatitis [27]. Notably, a recent international study of 299 patients with biopsy-confirmed MASH [28] and compensated cirrhosis, monitored for a median duration of 5 years, showed that those with T2DM had approximately twice the risk of death and liver-related outcomes, including hepatocellular carcinoma. This is consistent with our data, which showed that the median survival time of DM-MASH patients was shorter than that of patients with MASH without DM. Because HbA1c reflects metabolic status and may provide additional prognostic information in patients with liver cirrhosis, it could potentially be incorporated as a supplementary parameter in future assessments of hepatic reserve.

In diabetes, insulin function is impaired, leading to reduced glucose uptake by the muscles. Consequently, the energy supply becomes insufficient, muscle protein synthesis is suppressed, and muscle degradation is promoted, ultimately resulting in a decline in muscle mass [29]. In addition, diabetes induces systemic chronic inflammation and increases inflammatory cytokines, which further accelerate muscle breakdown and contribute to sarcopenia progression [30]. Moreover, prolonged hyperglycemia leads to the accumulation of advanced glycation end products, which deteriorate muscle protein quality. Furthermore, individuals with diabetes often adhere to dietary restrictions, leading to insufficient protein intake. Collectively, these factors contribute to sarcopenia progression in diabetic patients.

It was reported that T2DM and sarcopenia could have a bidirectional causal influence on each other [31,32], so sarcopenia can also be a risk factor for T2DM in older populations [33] Since muscle is a major tissue that consumes blood glucose, a decrease in muscle mass worsens blood glucose control, making diabetes more likely to progress. Furthermore, as sarcopenia advances, basal metabolism declines, leading to an increased risk of mortality. This also contributes to the worsening of insulin resistance. Additionally, the decline in muscle mass reduces physical performance, leading to decreased activity levels, which further complicates blood glucose management. A previous study found that 15 common genes and several shared pathways between sarcopenia and diabetes [34].

This study revealed that diabetes is a prognostic factor and a contributor to sarcopenia in patients with MASH-derived cirrhosis. Generally, liver fibrosis is considered an important prognostic factor in patients with MASH. Furthermore, the FIB-4 index was reported as a risk factor for hepatocarcinogenesis in patients with diabetes [35]. Therefore, liver fibrosis is undoubtedly another crucial factor. However, this study focused on patients with MASH-derived cirrhosis rather than MASH itself, meaning that liver fibrosis is already in its final stage. Meanwhile, diabetes and liver cirrhosis influence each other. The liver plays a crucial role in blood glucose regulation. As liver cirrhosis progresses, insulin resistance worsens, making diabetes management more challenging. Therefore, particular attention should be given to diabetes, particularly in patients with MASH-derived liver cirrhosis.

Nevertheless, this study has several limitations. First, it was a single-center, retrospective analysis, and is therefore subject to residual confounding and selection bias despite the application of multivariable adjustment. Several important covariates were not systematically captured, including antidiabetic therapies (GLP-1 receptor agonists, and SGLT2 inhibitors), nutritional intake, physical activity, inflammatory markers, and socioeconomic factors, which may have influenced the results. However, when survival was analyzed according to key factors of diabetes, including disease duration and insulin use, significant differences were observed. These factors were also associated with differences in the prevalence of sarcopenia.

Second, sarcopenia was diagnosed solely based on CT-derived skeletal muscle index (SMI). Future studies should incorporate functional assessments such as handgrip strength to provide a more comprehensive evaluation. Moreover, potential bias may exist given that only patients who underwent CT imaging were included. However, in Japan, cirrhosis patients typically undergo CT examinations every one to two years for hepatocellular carcinoma surveillance, which may have mitigated this bias.

Third, this was an exploratory study designed to identify potential prognostic factors in MASH cirrhosis; therefore, adjustments for multiple comparisons were not applied. Finally, we did not evaluate the severity of diabetes, endogenous insulin secretion, or treatment regimens. Future studies should clarify the relationship between diabetes and sarcopenia in MASH-related cirrhosis by incorporating these factors, including therapeutic strategies and insulin dynamics.

Finally, our study did not include a comparison group of patients with non-MASH cirrhosis. The inclusion of such a group would have provided valuable insights into whether the observed association between diabetes and sarcopenia is specific to MASH-related cirrhosis or reflects a more general feature of cirrhosis of diverse etiologies. However, the primary objective of this study was to focus on MASH cirrhosis and evaluate the impact of diabetes within this homogenous population. We acknowledge that comparing MASH with non-MASH etiologies represents an important area for future investigation.

## 5. Conclusions

In conclusion, we report that the presence of DM might increase the risk of sarcopenia-induced mortality in individuals with MASH-associated liver cirrhosis. Thus, DM can be used as a new additional diagnostic criterion to identify patients with poor prognoses among people with MASH-associated cirrhosis. In diabetic patients with MASH-derived liver cirrhosis, regular assessments of sarcopenia and strict blood glucose control should be emphasized. This study provides new insights into how diabetes plays a crucial role in patients with MASH-driven cirrhosis using clinical data and identifies the association between diabetes and sarcopenia.

## Figures and Tables

**Table 1:** Baseline characteristics of the patients with MASH in this study.

	Total	Non-DM Group	DM Group	*p*-Value
	(N = 169)	(N = 61)	(N = 108)	
**BMI (kg/m^2^)**	27.2 (19.2–40.4)	27.2 (16.9–45.6)	27.2 (18.3–46.3)	0.98
**waist (cm)**	94.6 (68–120)	93.9 (68–120)	95.0 (69–136.5)	0.57
**age (y)**	70 (44–98)	71 (44–98)	69.9 (47–89)	0.40
**male sex [n (%)]**	77 (45.5)	31(50.8)	46 (42.5)	0.22
**Child–Pugh class**				
**A**	111	39	80	
**B**	52	21	23	0.34
**C**	6	1	5	
**hemoglobin (g/dL)**	12.3 (7.7–16.2)	12.0 (8–16.2)	12.4 (6.9–17.3)	0.21
**platelet (×10^4^/µL)**	11.8 (3.5–30.3)	11.1 (3.4–21.7)	12.2 (3.5–32.3)	0.21
**AST (IU/L)**	46 (16–152)	43 (16–147)	47 (19–189)	0.35
**ALT (IU/L)**	35 (9–177)	30 (9–90)	38 (9–332)	0.35
**total protein (g/dL)**	7.0 (3.4–8.5)	6.8 (3.4–8.4)	7.1 (4.5–8.5)	0.04
**albumin (g/dL)**	3.7 (2.2–4.8)	3.6 (2.3–4.8)	3.7 (1.6–4.9)	0.35
**triglyceride (mg/dL)**	105 (10–465)	92 (10–236)	112 (25–465)	0.02
**total cholesterol (mg/dL)**	161 (40–347)	159 (40–249)	163 (69–347)	0.51
**cholinesterase (IU/L)**	214 (45–459)	202 (65–407)	221 (45–469)	0.15
**total bilirubin (mg/dL)**	1.3 (0.4–4.5)	1.3 (0.4–4.5)	1.3 (0.4–4.3)	0.66
**ammonia (µ/dL)**	50.8 (13.3–225.8)	55.9 (17–225.8)	48.0 (13.3–193.9)	0.14
**prothrombin time (%)**	78.9 (38–116)	77.5 (41–119)	79.7 (38–116)	0.37
**HbA1c (%)**	6.3 (3.9–11.1)	5.5 (3.9–6.4)	6.7 (4.5–11.1)	<0.001
**FIB-4 index**	4.8 (1.2–15.4)	4.6 (2–11.3)	4.9 (1.2–15.4)	0.49
**CT-SMI (cm^2^/m^2^)**				
**men**	49.0 (24–65.5)	49.9 (32.2–62.8)	46.5 (24–65.5)	0.50
**women**	40.5 (25–63)	44.3 (29.8–55)	41.0 (25–63.0)	0.53

DM, diabetes mellitus; AST, aspartate aminotransferase; ALT, alanine aminotransferase; BMI, body mass index; SMI, skeletal muscle index; Values are presented as average (range); Comparison of clinical characteristics among two groups. Statistical analysis was performed using the chi-square test or Mann–Whitney *U* test.

**Table 2:** Comparison of clinical characteristics between MASH patients with and without sarcopenia.

	Total	Non-Sarcopenia Group	Sarcopenia Group	*p*-Value
	(N = 169)	(N = 118)	(N = 51)	
**age (y)**	70 (44–98)	69.4 (44–98)	72.7 (50–88)	0.03
**male sex [n (%)]**	77 (45.5)	58 (49.1)	19 (37.2)	0.17
**Child–Pugh class**				
**A**	111	88	31	0.07
**B/C**	58	30	20	
**hemoglobin (g/dL)**	12.3 (7.7–16.2)	12.5 (7.7–17.3)	11.7 (6.9–17.2)	0.02
**platelet (×10^4^/µL)**	11.8 (3.5–30.3)	12.0 (3.4–32.3)	11.2 (4.2–23.8)	0.34
**AST (IU/L)**	46 (16–152)	45 (20–147)	48 (16–189)	0.51
**ALT (IU/L)**	35 (9–177)	34 (9–113)	38 (9–332)	0.57
**total protein (g/dL)**	7.0 (3.4–8.5)	7.1 (3.4–8.5)	6.9 (5.3–8.5)	0.16
**albumin (g/dL)**	3.7 (2.2–4.8)	3.7 (2.2–4.9)	3.5 (1.6–4.8)	0.03
**triglyceride (mg/dL)**	105 (10–465)	105 (10–257)	104 (25–465)	0.96
**total cholesterol (mg/dL)**	161 (40–347)	167 (40–347)	149 (69–272)	0.01
**cholinesterase (IU/L)**	214 (45–459)	226 (65–469)	187 (45–384)	0.006
**total bilirubin (mg/dL)**	1.3 (0.4–4.5)	1.35 (0.4–4.5)	1.3 (0.6–3.9)	0.72
**ammonia (µ/dL)**	50.8 (13.3–225.8)	52.2 (13.3–225.8)	47.8 (17–137.8)	0.39
**prothrombin time (%)**	78.9 (38–116)	80 (41–116)	76.6 (38–119)	0.22
**HbA1c (%)**	6.3 (3.9–11.1)	6.28 (3.9–11.1)	6.3 (4.5–9.1)	0.70
**FIB-4 index**	4.8 (1.2–15.4)	3.9 (1.32–13.6)	6.6 (1.24–15.4)	<0.001
**BMI (kg/m^2^)**	27.2 (19.2–40.4)	28.6 (16.9–46.3)	23.9 (18.3–33.2)	<0.001
**waist (cm)**	94.6 (68–120)	95.9 (69–136)	91.6 (68–136.5)	0.08
**CT-SMI (cm^2^/m^2^)**				
**men**	49.0 (24–65.5)	52.4 (42–65.5)	36.3 (24–41.7)	<0.001
**women**	40.5 (25–63)	47.8 (25–63)	34.2 (28.1–38)	<0.001
**diabetes, yes [n (%)]**	108 (63.9)	69 (58.5)	39 (76.5)	0.03

AST, aspartate aminotransferase; ALT, alanine aminotransferase; BMI, body mass index; SMI, skeletal muscle index; Values are presented as average (range); Comparison of clinical characteristics among two groups. Statistical analysis was performed using the chi-square test or Mann–Whitney *U* test.

**Table 3:** Predictors of sarcopenia in MASH patients with liver cirrhosis (N = 169).

	Univariate Analysis		Multivariate Analysis		Adjusted Analysis	
	OR (95%Cl)	*p*-Value	cOR (95%Cl)	*p*-Value	aOR (95%Cl)	*p*-Value
**a** **ge (y)**						
**<75**	1.00					
**≥75**	2.37 (1.21–4.64)	0.012	1.32 (0.52–3.35)	0.55		
**Child–Pugh class**						
**A**	1.00					
**B/C**	1.89 (0.94–3.80)	0.07	1.03 (0.26–4.05)	0.96		
**h** **emoglobin (g/dL)**						
**≥11.8**	1.00					
**<11.8**	2.47 (1.25–4.85)	0.008	0.52 (0.16–1.63)	0.26		
**a** **lbumin (g/dL)**						
**≥3.5**	1.00				1.00	
**<3.5**	2.21 (1.11–4.38)	0.023	1.58 (0.42–5.95)	0.49	2.16 (0.86–5.39)	0.09
**t** **otal cholesterol (mg/dL)**						
**≥144**	1.00					
**<144**	2.57 (1.30–5.06)	0.006	1.01 (0.36–2.76)	0.99		
**c** **holinesterase (IU/L)**						
**≥200**	1.00					
**<200**	1.7 (0.877–3.29)	0.11	0.52 (0.16–1.73)	0.29		
**FIB-4 index**						
**<5**	1.00				1.00	
**≥5**	7.74 (3.71–16.1)	<0.0001	8.6 (3.24–22.8)	<0.0001	8.1 (3.35–19.6)	<0.0001
**BMI (kg/m^2^)**						
**≥25**	1.00				1.00	
**<25**	0.112 (0.05–0.23)	<0.0001	0.13 (0.05–0.328)	<0.0001	0.114 (0.04–0.27)	<0.0001
**d** **iabetes**						
**n** **o**	1.00				1.00	
**yes**	2.3 (1.10–4.85)	0.02	4.38 (1.55–12.3)	0.005	3.67 (1.41–9.56)	<0.0001

BMI, body mass index; OR, odds ratio; cOR, crude OR; aOR, adjusted OR.

## Data Availability

The original contributions presented in this study are included in the article. Further inquiries can be directed to the corresponding author(s).

## Abbreviations

The following abbreviations are used in this manuscript:

BMI	Body mass index
CMRF	Cardiometabolic risk factor
CT	Computed tomography
DM	Diabetes mellitus
LRE	Liver-related events
MASLD	Metabolic dysfunction-associated steatotic liver disease
SMI	Skeletal muscle mass index
ALT	Alanine aminotransferase
AST	Aspartate aminotransferase
HCC	Hepatocellular carcinoma
MASH	Metabolic dysfunction-associated steatohepatitis
NAFLD	Nonalcoholic fatty liver disease
NASH	Nonalcoholic steatohepatitis
